# Supplementary material for: Hot spot prediction in protein-protein interactions by an ensemble system
Source: BMC Syst Biol. 2018 Dec 31;12(Suppl 9):132. doi: 10.1186/s12918-018-0665-8 (PMC6311905; doi:10.1186/s12918-018-0665-8)
Supplement: Supplementary file 2 — All relevant MATLAB code. A simple Matlab implement of our predictor. (ZIP 19,742 kb) [file 12918_2018_665_MOESM2_ESM.zip › Additional file 2/Read ASA/ASA Property/BID/1GL4A.txt]

 NetSurfP - Protein Surface Accessibility and Secondary Structure Predictions

  

|  |  |  |
| --- | --- | --- |
|  |  | NetSurfP - Protein Surface Accessibility and Secondary Structure Predictions  Technical University of Denmark |

  


---

```
# For publication of results, please cite:
# A generic method for assignment of reliability scores applied to solvent accessibility predictions.
# Bent Petersen, Thomas Nordahl Petersen, Pernille Andersen, Morten Nielsen and Claus Lundegaard
# BMC Structural Biology 2009, 9:51 doi:10.1186/1472-6807-9-51
#
# Column 1: Class assignment - B for buried or E for Exposed - Threshold: 25% exposure, but not based on RSA
# Column 2: Amino acid
# Column 3: Sequence name
# Column 4: Amino acid number
# Column 5: Relative Surface Accessibility - RSA
# Column 6: Absolute Surface Accessibility
# Column 7: Z-fit score for RSA prediction
# Column 8: Probability for Alpha-Helix
# Column 9: Probability for Beta-strand
# Column 10: Probability for Coil
E A  1GL4A                  1    0.835  92.072   0.503   0.003   0.003   0.994
E P  1GL4A                  2    0.476  67.573  -2.470   0.058   0.017   0.925
E L  1GL4A                  3    0.579 106.033  -2.128   0.109   0.005   0.886
E A  1GL4A                  4    0.542  59.772   0.401   0.246   0.004   0.750
E Q  1GL4A                  5    0.507  90.461   1.066   0.694   0.003   0.303
E Q  1GL4A                  6    0.497  88.746   0.186   0.600   0.003   0.397
B T  1GL4A                  7    0.225  31.180   0.468   0.622   0.015   0.363
B C  1GL4A                  8    0.044   6.178  -1.425   0.622   0.015   0.363
E A  1GL4A                  9    0.424  46.714   0.006   0.622   0.015   0.363
E N  1GL4A                 10    0.560  81.969  -0.087   0.430   0.016   0.555
E N  1GL4A                 11    0.430  62.893  -0.549   0.181   0.016   0.803
E R  1GL4A                 12    0.552 126.454  -0.199   0.058   0.017   0.925
E H  1GL4A                 13    0.502  91.241  -0.295   0.053   0.043   0.903
E Q  1GL4A                 14    0.484  86.389   0.910   0.053   0.043   0.903
B C  1GL4A                 15    0.037   5.125  -0.111   0.018   0.047   0.935
B S  1GL4A                 16    0.235  27.542   0.895   0.018   0.019   0.964
E V  1GL4A                 17    0.655 100.658   0.407   0.018   0.047   0.935
E H  1GL4A                 18    0.497  90.404   0.116   0.018   0.047   0.935
B A  1GL4A                 19    0.040   4.397  -0.571   0.019   0.141   0.840
E E  1GL4A                 20    0.450  78.685   1.082   0.022   0.552   0.426
B C  1GL4A                 21    0.036   4.998  -0.646   0.023   0.655   0.322
E R  1GL4A                 22    0.395  90.478   0.874   0.022   0.552   0.426
E D  1GL4A                 23    0.390  56.170  -0.677   0.020   0.205   0.775
E Y  1GL4A                 24    0.375  80.159   0.379   0.005   0.045   0.951
E A  1GL4A                 25    0.638  70.341  -1.595   0.005   0.015   0.979
E T  1GL4A                 26    0.531  73.691  -1.229   0.005   0.045   0.951
B G  1GL4A                 27    0.267  21.044  -1.540   0.004   0.197   0.799
B F  1GL4A                 28    0.173  34.801   0.788   0.004   0.616   0.381
E C  1GL4A                 29    0.386  54.251   0.952   0.001   0.900   0.099
B C  1GL4A                 30    0.024   3.356   0.028   0.001   0.900   0.099
E R  1GL4A                 31    0.400  91.554   1.248   0.001   0.900   0.099
B C  1GL4A                 32    0.077  10.811   0.032   0.003   0.718   0.279
E V  1GL4A                 33    0.490  75.267   0.703   0.021   0.279   0.699
E A  1GL4A                 34    0.716  78.936  -0.251   0.018   0.088   0.893
E N  1GL4A                 35    0.478  70.038  -1.734   0.018   0.047   0.935
B Y  1GL4A                 36    0.236  50.390  -0.462   0.019   0.141   0.840
E T  1GL4A                 37    0.516  71.625  -0.442   0.005   0.262   0.733
B G  1GL4A                 38    0.254  19.958  -2.721   0.004   0.197   0.799
E N  1GL4A                 39    0.590  86.420  -0.505   0.005   0.045   0.951
B G  1GL4A                 40    0.262  20.596  -2.010   0.018   0.088   0.893
E R  1GL4A                 41    0.408  93.386   1.518   0.022   0.552   0.426
E Q  1GL4A                 42    0.500  89.282   1.446   0.021   0.756   0.223
B C  1GL4A                 43    0.026   3.622  -0.076   0.023   0.655   0.322
E V  1GL4A                 44    0.335  51.566   0.705   0.021   0.451   0.528
E A  1GL4A                 45    0.650  71.608  -0.194   0.005   0.262   0.733
E E  1GL4A                 46    0.526  91.892  -0.319   0.005   0.045   0.951
E G  1GL4A                 47    0.654  51.462  -1.138   0.005   0.045   0.951
E S  1GL4A                 48    0.417  48.884  -0.504   0.004   0.138   0.858
B P  1GL4A                 49    0.235  33.304  -2.366   0.004   0.197   0.799
E Q  1GL4A                 50    0.458  81.781  -0.319   0.004   0.420   0.576
E R  1GL4A                 51    0.415  94.920  -0.755   0.004   0.514   0.481
B V  1GL4A                 52    0.278  42.698  -1.673   0.005   0.262   0.733
E N  1GL4A                 53    0.650  95.087  -1.540   0.004   0.085   0.910
B G  1GL4A                 54    0.264  20.753  -2.999   0.004   0.138   0.858
E K  1GL4A                 55    0.494 101.575   0.496   0.004   0.616   0.381
B V  1GL4A                 56    0.126  19.397  -1.166   0.002   0.816   0.182
E K  1GL4A                 57    0.458  94.108   0.617   0.002   0.816   0.182
B G  1GL4A                 58    0.124   9.759  -2.503   0.018   0.846   0.136
B R  1GL4A                 59    0.288  65.952  -0.172   0.021   0.756   0.223
B I  1GL4A                 60    0.084  15.632  -1.500   0.023   0.655   0.322
B F  1GL4A                 61    0.281  56.457  -0.687   0.021   0.451   0.528
B V  1GL4A                 62    0.289  44.465  -0.962   0.021   0.279   0.699
E G  1GL4A                 63    0.394  30.968  -1.655   0.018   0.088   0.893
E S  1GL4A                 64    0.587  68.761  -2.155   0.019   0.141   0.840
E S  1GL4A                 65    0.317  37.094  -0.066   0.022   0.359   0.619
E Q  1GL4A                 66    0.613 109.535  -0.877   0.021   0.451   0.528
B V  1GL4A                 67    0.195  30.033  -0.072   0.020   0.205   0.775
E P  1GL4A                 68    0.351  49.835  -1.227   0.020   0.205   0.775
B V  1GL4A                 69    0.209  32.062  -0.355   0.021   0.451   0.528
E V  1GL4A                 70    0.428  65.707   0.151   0.022   0.552   0.426
B F  1GL4A                 71    0.205  41.063  -0.593   0.022   0.552   0.426
E E  1GL4A                 72    0.436  76.204  -0.745   0.021   0.451   0.528
E N  1GL4A                 73    0.587  85.922  -0.867   0.022   0.359   0.619
B T  1GL4A                 74    0.152  21.027   0.251   0.004   0.514   0.481
E D  1GL4A                 75    0.408  58.735   0.408   0.003   0.718   0.279
B L  1GL4A                 76    0.164  30.065   0.043   0.002   0.816   0.182
B H  1GL4A                 77    0.191  34.688   0.084   0.021   0.756   0.223
B S  1GL4A                 78    0.190  22.256  -1.296   0.023   0.655   0.322
B Y  1GL4A                 79    0.235  50.326  -0.218   0.021   0.756   0.223
B V  1GL4A                 80    0.250  38.440  -0.849   0.018   0.846   0.136
B V  1GL4A                 81    0.187  28.757  -0.606   0.021   0.756   0.223
B M  1GL4A                 82    0.315  63.072  -1.365   0.021   0.451   0.528
E N  1GL4A                 83    0.655  95.951  -2.049   0.019   0.141   0.840
E H  1GL4A                 84    0.494  89.804  -1.257   0.018   0.088   0.893
E G  1GL4A                 85    0.333  26.168  -0.967   0.021   0.279   0.699
B R  1GL4A                 86    0.251  57.502   0.454   0.018   0.846   0.136
B S  1GL4A                 87    0.105  12.247   0.227   0.011   0.918   0.071
B Y  1GL4A                 88    0.148  31.606   1.052   0.011   0.918   0.071
B T  1GL4A                 89    0.082  11.318  -0.080   0.011   0.918   0.071
B A  1GL4A                 90    0.144  15.880   0.066   0.011   0.918   0.071
B I  1GL4A                 91    0.077  14.264  -0.783   0.023   0.655   0.322
B S  1GL4A                 92    0.217  25.444  -0.199   0.021   0.279   0.699
E T  1GL4A                 93    0.516  71.541   0.307   0.052   0.084   0.864
B I  1GL4A                 94    0.126  23.236   0.780   0.053   0.043   0.903
E P  1GL4A                 95    0.297  42.130   0.156   0.115   0.016   0.868
E E  1GL4A                 96    0.649 113.293  -0.100   0.321   0.003   0.675
E T  1GL4A                 97    0.532  73.788  -0.715   0.321   0.003   0.675
B V  1GL4A                 98    0.112  17.261  -0.077   0.502   0.002   0.495
B G  1GL4A                 99    0.092   7.240  -0.451   0.502   0.002   0.495
E Y  1GL4A                100    0.381  81.334   0.351   0.694   0.003   0.303
B S  1GL4A                101    0.171  20.006  -1.248   0.694   0.003   0.303
B L  1GL4A                102    0.060  11.004   0.353   0.782   0.003   0.216
B L  1GL4A                103    0.246  45.079   0.400   0.802   0.014   0.185
B P  1GL4A                104    0.198  28.096   0.032   0.858   0.002   0.139
B L  1GL4A                105    0.065  11.883   0.150   0.858   0.002   0.139
B A  1GL4A                106    0.147  16.254   0.348   0.858   0.002   0.139
B P  1GL4A                107    0.164  23.314  -1.392   0.802   0.014   0.185
B I  1GL4A                108    0.121  22.440  -1.141   0.802   0.014   0.185
B G  1GL4A                109    0.184  14.504  -1.487   0.717   0.014   0.269
B G  1GL4A                110    0.174  13.662  -1.010   0.622   0.015   0.363
B I  1GL4A                111    0.145  26.880  -0.818   0.717   0.014   0.269
B I  1GL4A                112    0.105  19.333   0.165   0.802   0.014   0.185
B G  1GL4A                113    0.134  10.514   0.098   0.831   0.044   0.125
B W  1GL4A                114    0.087  20.899  -0.063   0.831   0.044   0.125
B M  1GL4A                115    0.083  16.568  -0.264   0.751   0.050   0.199
B F  1GL4A                116    0.110  22.157   0.369   0.701   0.107   0.192
B A  1GL4A                117    0.076   8.364  -0.605   0.502   0.102   0.396
E V  1GL4A                118    0.294  45.126   0.723   0.307   0.165   0.527
E E  1GL4A                119    0.452  78.982  -0.170   0.216   0.235   0.548
E Q  1GL4A                120    0.395  70.601   0.108   0.113   0.087   0.800
E D  1GL4A                121    0.584  84.198  -1.686   0.053   0.043   0.903
E G  1GL4A                122    0.685  53.886  -0.499   0.053   0.043   0.903
B F  1GL4A                123    0.168  33.657  -1.055   0.056   0.142   0.802
E K  1GL4A                124    0.387  79.668  -0.067   0.064   0.216   0.721
B N  1GL4A                125    0.192  28.123  -1.134   0.064   0.216   0.721
B G  1GL4A                126    0.114   8.964  -1.409   0.021   0.279   0.699
B F  1GL4A                127    0.142  28.459   0.122   0.021   0.756   0.223
B S  1GL4A                128    0.258  30.226   0.315   0.011   0.918   0.071
B I  1GL4A                129    0.100  18.444   0.312   0.018   0.846   0.136
B T  1GL4A                130    0.247  34.245  -0.332   0.004   0.514   0.481
E G  1GL4A                131    0.538  42.333  -0.983   0.004   0.138   0.858
B G  1GL4A                132    0.172  13.544  -1.383   0.005   0.262   0.733
E E  1GL4A                133    0.489  85.358   0.855   0.002   0.816   0.182
B F  1GL4A                134    0.142  28.439   0.265   0.001   0.900   0.099
E T  1GL4A                135    0.439  60.834   1.095   0.001   0.900   0.099
B R  1GL4A                136    0.222  50.815   0.801   0.001   0.900   0.099
E Q  1GL4A                137    0.506  90.354   1.019   0.001   0.959   0.040
B A  1GL4A                138    0.108  11.891   0.368   0.001   0.959   0.040
E E  1GL4A                139    0.367  64.150   0.477   0.001   0.959   0.040
B V  1GL4A                140    0.056   8.623   0.931   0.001   0.959   0.040
E T  1GL4A                141    0.386  53.524   0.754   0.000   0.983   0.017
B F  1GL4A                142    0.114  22.840   0.285   0.001   0.959   0.040
E L  1GL4A                143    0.321  58.775   0.661   0.004   0.420   0.576
E G  1GL4A                144    0.478  37.642  -0.856   0.005   0.045   0.951
E H  1GL4A                145    0.544  98.972  -1.456   0.005   0.015   0.979
E P  1GL4A                146    0.593  84.118  -0.876   0.004   0.138   0.858
B G  1GL4A                147    0.270  21.288  -0.236   0.003   0.718   0.279
E K  1GL4A                148    0.360  73.990   1.271   0.001   0.900   0.099
B L  1GL4A                149    0.043   7.873   0.624   0.001   0.959   0.040
E V  1GL4A                150    0.311  47.770   1.495   0.001   0.959   0.040
B L  1GL4A                151    0.047   8.532   0.782   0.001   0.959   0.040
E K  1GL4A                152    0.350  71.933   1.850   0.011   0.918   0.071
B Q  1GL4A                153    0.114  20.414   0.282   0.011   0.918   0.071
E Q  1GL4A                154    0.482  86.014   0.981   0.011   0.918   0.071
B F  1GL4A                155    0.105  21.033  -0.218   0.003   0.718   0.279
E S  1GL4A                156    0.495  58.037   0.196   0.004   0.420   0.576
E G  1GL4A                157    0.354  27.883  -1.455   0.004   0.197   0.799
B I  1GL4A                158    0.250  46.287   0.013   0.004   0.085   0.910
B D  1GL4A                159    0.236  34.051   0.202   0.005   0.045   0.951
E E  1GL4A                160    0.537  93.744  -0.850   0.052   0.084   0.864
E H  1GL4A                161    0.502  91.332  -0.839   0.018   0.088   0.893
B G  1GL4A                162    0.218  17.141  -1.089   0.004   0.138   0.858
B H  1GL4A                163    0.152  27.722   0.286   0.004   0.616   0.381
B L  1GL4A                164    0.047   8.569  -0.191   0.002   0.816   0.182
B T  1GL4A                165    0.125  17.407   0.199   0.001   0.900   0.099
B I  1GL4A                166    0.049   9.084   0.205   0.001   0.900   0.099
B S  1GL4A                167    0.255  29.851   0.583   0.001   0.900   0.099
B T  1GL4A                168    0.055   7.615  -0.080   0.001   0.900   0.099
E E  1GL4A                169    0.282  49.178   0.348   0.001   0.959   0.040
B L  1GL4A                170    0.084  15.454  -0.304   0.001   0.959   0.040
E E  1GL4A                171    0.438  76.536   0.162   0.003   0.718   0.279
B G  1GL4A                172    0.143  11.246  -1.466   0.004   0.514   0.481
E R  1GL4A                173    0.429  98.218   0.244   0.004   0.616   0.381
B V  1GL4A                174    0.122  18.705   0.735   0.021   0.451   0.528
B P  1GL4A                175    0.203  28.834   0.379   0.020   0.205   0.775
E Q  1GL4A                176    0.529  94.444  -1.027   0.020   0.205   0.775
B I  1GL4A                177    0.127  23.587   0.331   0.019   0.141   0.840
E P  1GL4A                178    0.281  39.860  -0.138   0.018   0.088   0.893
E Y  1GL4A                179    0.538 114.992  -0.742   0.018   0.019   0.964
E G  1GL4A                180    0.668  52.611  -1.030   0.018   0.019   0.964
B A  1GL4A                181    0.168  18.558  -0.963   0.004   0.085   0.910
E S  1GL4A                182    0.586  68.714   1.041   0.004   0.514   0.481
B V  1GL4A                183    0.107  16.507   0.426   0.003   0.718   0.279
E H  1GL4A                184    0.499  90.768   1.145   0.018   0.846   0.136
B I  1GL4A                185    0.116  21.478   0.702   0.023   0.655   0.322
E E  1GL4A                186    0.597 104.243   1.069   0.020   0.205   0.775
E P  1GL4A                187    0.623  88.418   0.695   0.018   0.047   0.935
B Y  1GL4A                188    0.176  37.611   0.709   0.020   0.205   0.775
E T  1GL4A                189    0.555  76.937   0.668   0.022   0.552   0.426
E E  1GL4A                190    0.340  59.450   0.034   0.021   0.756   0.223
E L  1GL4A                191    0.435  79.667   0.793   0.021   0.756   0.223
B Y  1GL4A                192    0.144  30.666   1.210   0.021   0.756   0.223
E H  1GL4A                193    0.355  64.520  -0.032   0.021   0.756   0.223
B Y  1GL4A                194    0.295  63.106   0.029   0.004   0.514   0.481
E S  1GL4A                195    0.382  44.817  -0.710   0.004   0.197   0.799
E S  1GL4A                196    0.594  69.582  -1.101   0.005   0.045   0.951
E S  1GL4A                197    0.320  37.539  -2.446   0.004   0.197   0.799
E V  1GL4A                198    0.283  43.451   0.543   0.002   0.816   0.182
B I  1GL4A                199    0.083  15.281   0.035   0.001   0.900   0.099
B T  1GL4A                200    0.266  36.866   0.571   0.001   0.900   0.099
B S  1GL4A                201    0.058   6.774  -1.106   0.001   0.900   0.099
B S  1GL4A                202    0.242  28.409  -0.058   0.002   0.816   0.182
B S  1GL4A                203    0.124  14.568  -0.940   0.002   0.816   0.182
B T  1GL4A                204    0.221  30.667  -0.442   0.003   0.718   0.279
B R  1GL4A                205    0.149  34.213  -0.361   0.003   0.718   0.279
E E  1GL4A                206    0.290  50.593  -0.315   0.002   0.816   0.182
B Y  1GL4A                207    0.069  14.809   0.502   0.001   0.900   0.099
E T  1GL4A                208    0.249  34.564   0.436   0.001   0.959   0.040
B V  1GL4A                209    0.054   8.238  -0.005   0.001   0.900   0.099
E M  1GL4A                210    0.327  65.413  -0.175   0.018   0.846   0.136
B E  1GL4A                211    0.210  36.739  -0.291   0.023   0.655   0.322
E P  1GL4A                212    0.359  50.871  -1.097   0.021   0.279   0.699
E D  1GL4A                213    0.495  71.358  -1.197   0.019   0.141   0.840
E Q  1GL4A                214    0.553  98.855  -1.068   0.019   0.141   0.840
E D  1GL4A                215    0.682  98.262  -2.048   0.018   0.088   0.893
E G  1GL4A                216    0.533  41.923  -2.091   0.018   0.088   0.893
E A  1GL4A                217    0.469  51.673  -2.033   0.019   0.141   0.840
E A  1GL4A                218    0.378  41.656  -0.867   0.005   0.336   0.660
E P  1GL4A                219    0.496  70.326  -0.362   0.004   0.420   0.576
E S  1GL4A                220    0.470  55.049  -0.891   0.004   0.514   0.481
E H  1GL4A                221    0.338  61.428   0.152   0.003   0.718   0.279
E T  1GL4A                222    0.449  62.207  -0.666   0.002   0.816   0.182
B H  1GL4A                223    0.178  32.451   0.964   0.001   0.900   0.099
E I  1GL4A                224    0.347  64.176  -0.340   0.001   0.959   0.040
B Y  1GL4A                225    0.091  19.404   0.589   0.001   0.900   0.099
E Q  1GL4A                226    0.356  63.671   0.811   0.001   0.959   0.040
B W  1GL4A                227    0.108  26.022   0.709   0.001   0.900   0.099
E R  1GL4A                228    0.449 102.798   0.440   0.002   0.816   0.182
B Q  1GL4A                229    0.091  16.324   0.501   0.002   0.816   0.182
E T  1GL4A                230    0.387  53.732   0.579   0.001   0.900   0.099
B I  1GL4A                231    0.064  11.748   1.175   0.001   0.900   0.099
E T  1GL4A                232    0.302  41.860   1.528   0.001   0.959   0.040
B F  1GL4A                233    0.094  18.846   0.880   0.018   0.846   0.136
E Q  1GL4A                234    0.512  91.461   0.701   0.023   0.655   0.322
E E  1GL4A                235    0.372  64.919  -0.844   0.021   0.451   0.528
B C  1GL4A                236    0.086  12.074   0.068   0.021   0.451   0.528
E A  1GL4A                237    0.489  53.833   0.227   0.020   0.205   0.775
E H  1GL4A                238    0.407  74.088  -0.941   0.018   0.047   0.935
E D  1GL4A                239    0.548  78.952  -0.301   0.053   0.043   0.903
E D  1GL4A                240    0.561  80.768  -0.939   0.058   0.017   0.925
E A  1GL4A                241    0.421  46.394  -1.243   0.053   0.043   0.903
E R  1GL4A                242    0.546 125.057  -0.345   0.053   0.043   0.903
E P  1GL4A                243    0.409  58.009  -0.768   0.018   0.019   0.964
E A  1GL4A                244    0.595  65.624  -1.130   0.018   0.047   0.935
B L  1GL4A                245    0.185  33.819   0.598   0.053   0.043   0.903
E P  1GL4A                246    0.434  61.585   0.019   0.018   0.019   0.964
E S  1GL4A                247    0.370  43.376   0.056   0.064   0.216   0.721
B T  1GL4A                248    0.239  33.135  -0.753   0.079   0.592   0.329
B Q  1GL4A                249    0.220  39.221   0.026   0.021   0.756   0.223
E Q  1GL4A                250    0.352  62.814   1.149   0.018   0.846   0.136
B L  1GL4A                251    0.066  12.140   0.775   0.018   0.846   0.136
E S  1GL4A                252    0.345  40.387   0.908   0.018   0.846   0.136
B V  1GL4A                253    0.078  12.019  -0.628   0.021   0.756   0.223
E D  1GL4A                254    0.389  56.084  -0.180   0.021   0.756   0.223
E S  1GL4A                255    0.303  35.535   0.186   0.085   0.775   0.140
B V  1GL4A                256    0.101  15.493  -0.202   0.085   0.775   0.140
B F  1GL4A                257    0.217  43.492   0.169   0.085   0.775   0.140
B V  1GL4A                258    0.111  17.061  -0.978   0.087   0.683   0.230
B L  1GL4A                259    0.194  35.485  -0.613   0.023   0.655   0.322
B Y  1GL4A                260    0.167  35.667   0.193   0.022   0.552   0.426
E N  1GL4A                261    0.305  44.623   0.751   0.021   0.279   0.699
E K  1GL4A                262    0.500 102.871   0.012   0.113   0.087   0.800
E E  1GL4A                263    0.582 101.710  -0.128   0.268   0.043   0.689
E E  1GL4A                264    0.445  77.811  -0.095   0.354   0.048   0.598
E R  1GL4A                265    0.408  93.546   0.703   0.386   0.097   0.517
B I  1GL4A                266    0.082  15.078   0.385   0.373   0.401   0.226
B L  1GL4A                267    0.071  13.055   0.485   0.273   0.587   0.140
B R  1GL4A                268    0.226  51.685   0.631   0.265   0.667   0.069
B Y  1GL4A                269    0.094  20.088   0.394   0.265   0.667   0.069
B A  1GL4A                270    0.083   9.136  -0.097   0.265   0.667   0.069
B L  1GL4A                271    0.067  12.176  -0.665   0.172   0.690   0.138
B S  1GL4A                272    0.209  24.553   0.130   0.169   0.612   0.219
B N  1GL4A                273    0.152  22.194  -0.746   0.066   0.296   0.638
E S  1GL4A                274    0.371  43.458  -0.199   0.021   0.279   0.699
B I  1GL4A                275    0.100  18.463  -1.112   0.022   0.359   0.619
B G  1GL4A                276    0.172  13.505  -0.778   0.020   0.205   0.775
B P  1GL4A                277    0.217  30.849  -0.108   0.018   0.047   0.935
E V  1GL4A                278    0.390  59.897  -1.018   0.018   0.019   0.964
E R  1GL4A                279    0.699 160.094  -2.006   0.016   0.005   0.979
E D  1GL4A                280    0.677  97.599  -2.405   0.016   0.005   0.979
E G  1GL4A                281    0.421  33.109  -1.895   0.005   0.015   0.979
E S  1GL4A                282    0.577  67.683  -0.099   0.005   0.015   0.979
E P  1GL4A                283    0.390  55.384  -1.910   0.018   0.019   0.964
E D  1GL4A                284    0.677  97.512  -1.571   0.018   0.019   0.964
E A  1GL4A                285    0.847  93.339  -0.697   0.003   0.003   0.994

Explain the output.  Go back.
```

---
